# Ecological, Social and Biological Risk Factors for Continued *Trypanosoma cruzi* Transmission by *Triatoma dimidiata* in Guatemala

**DOI:** 10.1371/journal.pone.0104599

**Published:** 2014-08-29

**Authors:** Dulce M. Bustamante, Sandra M. De Urioste-Stone, José G. Juárez, Pamela M. Pennington

**Affiliations:** 1 Department of Biology, San Carlos University, Guatemala City, Guatemala; 2 School of Forest Resources, University of Maine, Orono, Maine, United States of America; 3 Health Studies Center, Universidad del Valle de Guatemala, Guatemala City, Guatemala; University of Tours, France

## Abstract

**Background:**

Chagas disease transmission by *Triatoma dimidiata* persists in Guatemala and elsewhere in Central America under undefined ecological, biological and social (eco-bio-social) conditions.

**Methodology:**

Eco-bio-social risk factors associated with persistent domiciliary infestation were identified by a cross-sectional survey and qualitative participatory methods. Quantitative and qualitative data were generated regarding *Trypanosoma cruzi* reservoirs and triatomine hosts. Blood meal analysis and infection of insects, dogs and rodents were determined. Based on these data, multimodel inference was used to identify risk factors for domestic infestation with the greatest relative importance (>0.75).

**Principal Findings:**

Blood meal analysis showed that 64% of 36 bugs fed on chickens, 50% on humans, 17% on dogs; 24% of 34 bugs fed on *Rattus rattus* and 21% on *Mus musculus*. Seroprevalence among 80 dogs was 37%. Eight (17%) of 46 *M. musculus* and three (43%) of seven *R. rattus* from households with infected triatomines were infected with *T. cruzi* Distinct Typing Unit I. Results from interviews and participatory meetings indicated that vector control personnel and some householders perceived chickens roosting and laying eggs in the house as bug infestation risk factors. House construction practices were seen as a risk factor for bug and rodent infestation, with rodents being perceived as a pest by study participants. Multimodel inference showed that house infestation risk factors of high relative importance are dog density, mouse presence, interior wall plaster condition, dirt floor, tile roofing and coffee tree presence.

**Conclusions/Significance:**

Persistent house infestation is closely related to eco-bio-social factors that maintain productive *T. dimidiata* habitats associated with dogs, chickens and rodents. Triatomine, dog and rodent infections indicate active *T. cruzi* transmission. Integrated vector control methods should include actions that consider the role of peridomestic animals in transmission and community memberś level of knowledge, attitudes and practices associated with the disease and transmission process.

## Introduction

Chagas disease is a vector-borne neglected tropical disease that continues to affect the most vulnerable populations across Latin America. Integrated vector management (IVM) was proposed by the World Health Organization (WHO) as part of the 2008–2015 strategy to control neglected tropical diseases [1]. Tropical Disease Research/WHO implemented an initiative to study the ecological, biological and social (eco-bio-social) factors that lead to the presence of dengue and Chagas disease vectors under different eco-epidemiological settings at the local level [2], [3]. The local ecology of vector-borne diseases must be considered by epidemiologists, public health officials and policy makers in the development of novel IVM and disease control strategies.

Despite advances in the control of Chagas disease vectors, insecticide-based control is limited by local conditions that lead to persistent infestation foci, sometimes derived from peridomestic habitats [4]–[8]. The transmission cycle includes mammalian reservoirs of the parasite and triatomine species, such as *Triatoma dimidiata* and *Triatoma infestans*, which colonize domestic and peridomestic environments [6], [9]. Indoor residual insecticide spraying and changes in house construction methods are the only public health vector control tools currently available. Meanwhile, unidentified peridomestic habitats persist as a vector control challenge.

In Central America, a Chagas disease control initiative was launched in 2001 based largely on indoor residual spraying with insecticides [5]. Entomological surveys at the Municipality geopolitical level were performed in Guatemala to determine vector distribution. These surveys were followed by the implementation of a National Chagas Disease Control Program that applied residual pyrethroid insecticides in all affected Municipalities. Evaluations of the initiative showed that *T. dimidiata* infestation levels were reduced up to nine-fold in many Municipalities [5]. However, despite multiple insecticide applications, infestation levels remained clustered in some communities [8], [10], [11].

Chagas disease eco-epidemiology is related to local environmental conditions combined with socio-economic and cultural factors that lead to the presence of animal nests harboring triatomines near or within households [3], [9], [12], [13]. Dogs, cats and rodents are synanthropic reservoirs of *T. cruzi* and common blood meal sources [14]–[16]. Chickens are a blood source for various triatomines and are thought to play a role in house colonization, even though they are refractory to *T. cruzi* [17], [18]. Thus, the definition of risk factors related to vector infestation and parasite transmission should consider the eco-bio-social factors that lead to the presence of blood meal sources and reservoir hosts in and around the household.

This study used a multidisciplinary mixed methods approach to identify eco-bio-social factors of persistent triatomine intradomiciliary infestation in a region of Guatemala where community-wide insecticide applications were less effective compared to other areas of the country.

## Methods

### Ethics statement

The study protocol was approved by the Universidad del Valle de Guatemala (IRB 00002049, FWA 00001902) and World Health Organization Institutional Review Boards. Individual written informed consent was obtained from study participants before household surveys and group written consent was obtained before each group meeting. Consents included permission to take photographs and make video recordings of activities. This study was performed in strict accordance with the recommendations in the Guide for the Care and Use of Laboratory Animals of the National Institutes of Health. The protocol was approved by the Institutional Animal Care and Use Committee of the Universidad del Valle de Guatemala (AWLAW No. A5847-01).

### Study area and population

**Figure 1:**
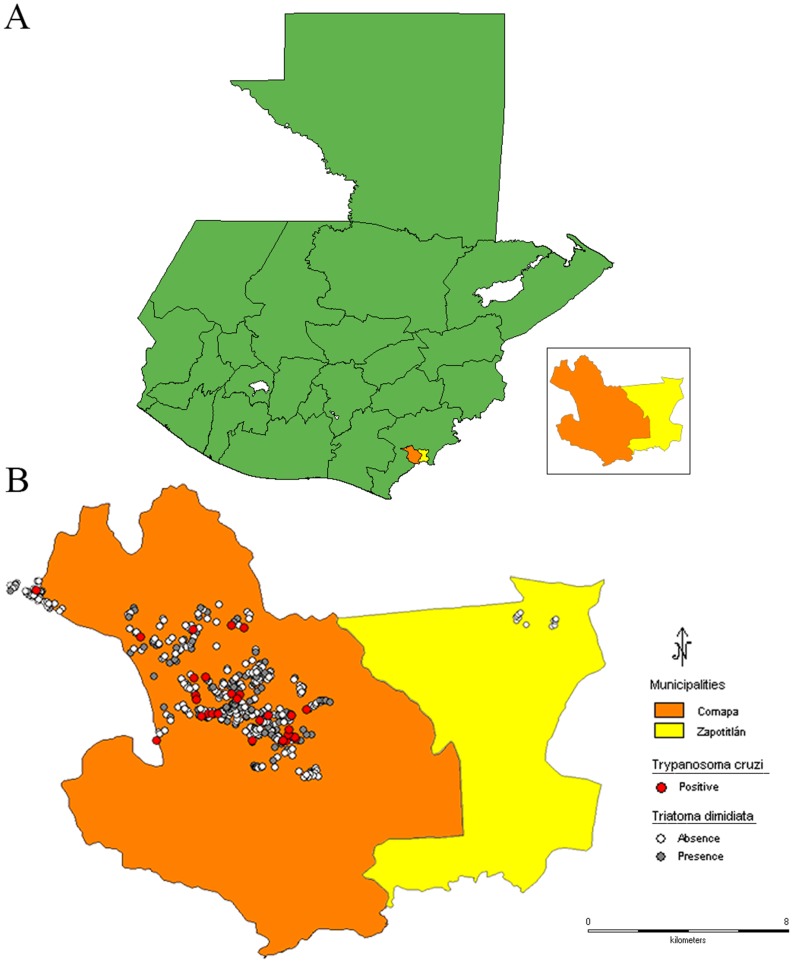
Study site location and household infestation status in 2011. **A**. Map of Guatemala showing the location of Comapa and Zapotitlán, Jutiapa. **B**. Map of Comapa and Zapotitlán showing the presence (grey circles) and absence (white circles) of household infestation of *Triatoma dimidiata*, and triatomines infected with *Trypanosoma cruzi* (red circle). No triatomines were collected in the municipality of Zapotitlán. *Because of conflicts between municipalities three communities considered part of Comapa are located outside the map boundary.

This study was conducted in the municipalities of Comapa and Zapotitlán, department of Jutiapa, in eastern Guatemala (Figure 1). Comapa is located at: −89°54′46.8″ and 14°6′38.6748″, and Zapotitlán at −89° 49′ 33.1314″ and 14° 8′ 15.1866″. More than 80% of the populations of Comapa and Zapotitlán live in rural areas, more than 80% live in poverty and more than 30% live in extreme poverty [19].

### Study design

A multilevel triangulation mixed methods design was used to converge and validate quantitative results and qualitative information [20] from multiple disciplines. This design is used to address different levels within an eco-bio-social system; each method is treated as one level or data layer. Quantitative and qualitative methods from diverse fields are utilized. Findings from all levels are integrated for an overall interpretation. A combination of biophysical and social science research methods were used including: household and entomological surveys, rodent survey, canine serological survey, qualitative data collection methods (semi-structured interviews and participatory community meetings).

A cross-sectional study was performed between January and February 2011 among randomly selected villages, to produce a baseline survey for entomological, ecological and social factors. A follow-up animal/entomological survey was undertaken in a subset of the houses from March to July 2011, including canine serological and rodent surveys at the household level. Interviews were conducted between January and August 2011. Participatory group meetings were held with selected communities from March to May 2011.

### Community selection and sample size

Baseline survey communities were selected based on location, >800 meters above sea level, >20% baseline infestation prevalence for *T. dimidiata* (i.e. before the Ministry of Health control program in 2004–2010) and a history of multiple insecticide applications with persistent infestation by this vector [11]. A total of 40 communities were identified, with a total of 3,944 households (population). Thirty-two communities (30 from Comapa and two from Zapotitlán) were randomly selected with two-stage cluster sampling that considered probability proportional to size of clusters and systematic probability sampling of households [21]–[23].

The first stage used a probability proportional to size selection of clusters since the number of elements in each community was not the same [21]–[23]; clusters were selected based on a geo-referenced sampling frame of communities with ArcMap 9.3.1 (ESRI, Redlands, CA, USA). Fifty-nine clusters of eight households each were selected. To reduce selection/interviewer error in the field, the second stage included systematic probability sampling [21] to randomly select households from the geo-referenced sampling frame using ArcMap 9.3.1. For each selected community an interval was estimated using the number of households to be sampled (eight, 16, 24, 32, or 40) and the total population of households for that community; in addition a random start was chosen for each community [21]. Once the interval and random start were selected, a systematic random sampling of households was performed for each community, utilizing ArcMap 9.3.1. Maps with randomly selected households were printed for each community. In the field, selected houses where identified and visited to conduct the household demographic and Knowledge Attitude and Practices (KAP) questionnaire and entomological surveys. In the case a household previously selected was not found, interviewers searched the next house to the right on the direction previously established.

A follow-up animal survey included a subset of 23 randomly selected communities (all were from Comapa due to random selection) and 248 systematically selected households surveyed at baseline. In two cases, the originally sampled house was not available for the follow-up survey and a new household was systematically recruited. Households without an adult at the time of the survey were not eligible for the study. Each household was georeferenced with a geographical positioning device (Garmin, Schaffhausen, Switzerland).

The sample size was 472 households for the baseline survey and 248 households for the follow-up survey. The baseline survey sample assumed a 20% infestation with 7% precision, and a 0% false-negative error rate, for a design effect of 2. The sample allowed detection of risk factors with a prevalence of 20% to 80% and a minimum detectable odds ratio (OR) of 2 for logistic regression with a single binary covariate, according to the Wald test [24], [25]. Standard logistic regressions are likely underestimating both effect-sizes (ORs) and their variances. The follow-up survey sample size was based on the expected 30% dog seroprevalence and number of households reporting dog ownership at baseline.

### Household and entomological surveys

To characterize households, a structured questionnaire was used for a face-to-face household demographic and KAP survey [26], according to Dillman's Tailored Design Method [27]. The questionnaire consisted of closed-ended, semiclosed-ended and ranking items related to Chagas disease, triatomines, animal management, rodents, household construction materials and peridomestic structures. The questionnaire was validated in the field previous to its implementation.

For the entomological survey during the baseline and follow-up surveys, each house was searched by two members of the team in the domestic and peridomestic environments (15 min each ecotope) using the person-hour method [28]. Triatomine presence, abundance and location were recorded. Specimens were transported to the laboratory for processing.

During the follow-up animal survey, householders were asked to search for triatomines during three consecutive nights to gain an understanding of community participation in triatomine surveillance. Given that the collection ecotope was not noted, the results of this participant entomological survey were not used in univariate analyses nor model generation.

Descriptive statistics and normality tests were conducted on all variables from the KAP surveys. According to the results from the descriptive tests, level of measurement and normality tests, contingency tables, chi-squared, phi, Cramer's V and odds ratios were used to compare all variables from the KAP surveys against the entomological variable of triatomine presence in domestic environments as determined by the person-hour method (triatomines collected in peridomestic environments were not included in these analyses). The p value was set at 0.05. Phi values of 0.001 to 0.10 indicated weak association, and values between 0.11 and 0.30 indicated moderate associations [29].

### Triatomine infection and blood meal analysis

Bias in triatomine processing was introduced by a mortality during transport of 477 (71%) of 669 bugs, due to high environmental temperatures. For each household, live bugs were randomly selected for dissection. A rectal puncture was analyzed by screening 20 fields at 40× by phase contrast microscopy. Individual live bugs from each household were screened until the first positive specimen was identified. Live and dead bugs were preserved in 95% ethanol for molecular analysis. Midgut DNA was extracted from all triatomines (n = 192) that arrived alive at the laboratory and from 9 dead specimens. Triatomine midguts were dissected from ethanol-preserved specimens and air dried; then 25–50 mg was homogenized with a metal bead in 600 µL DNAzol (Invitrogen, Carlsbad, CA, USA) on a Mix Mill MM 400 homogenizer (Retsch, Haan, Germany). After overnight incubation at 4°C, 400 µL DNAzol was added, followed by incubation for 1 h at room temperature and centrifugation at 13,000 g for 15 min at room temperature. The DNA was precipitated with 500 µL absolute ethanol and washed twice with 70% ethanol. All extracted samples (n = 201) were screened with a universal vertebrate mitochondrial cytochrome *b* (cyt *b*) PCR (Table S1 in File S1). Samples were run on 2% agarose/1×Tris acetic acid EDTA buffer gels containing ethidium bromide (Promega, Madison, WI,USA) and visualized under ultraviolet light (UVP, Upland, CA, USA). Thirty six samples positive for universal cyt *b* were subjected to additional individual PCR with specific primers for *Homo sapiens* (β-globin), *Gallus gallus*, *Canis familiaris, Rattus rattus* and *Mus musculus*. Polymerase chain reaction and fragment size analysis for *T. cruzi* minicircle was performed on β-globin positive triatomines (Table S1 in File S1).

### Rodent survey and tissue processing

During the follow-up survey, rodent infestation was estimated for the subset of houses by placing five 7.6×8.9×22.9 cm Sherman traps inside each house for three consecutive nights with daily checking. Captured animals were sedated with pentobarbital and euthanized by cardiac puncture followed by cervical dislocation. A blood smear was prepared and heart tissue was collected in 95% ethanol. The carcass was preserved in ethanol and transported to the laboratory for taxonomic characterization. All preserved rodent hearts were analyzed for *T. cruzi* DNA from 15 of 17 households with at least one microscopically positive triatomine. Polymerase chain reaction for *T. cruzi* characterization was performed on rodent heart DNA extracted as described above. Selected samples were typed at the Distinct Typing Unit level by glucose-6-phosphate isomerase (GPI) sequence analysis. The PCR product of GPI was obtained according to conditions in Table S1 in File S1, purified with a Wizard PCR purification kit (Promega, Winsconsin, USA) and sequenced at Macrogen, Inc. (Rockville, MD, USA). The remainder of the samples were screened for *T. cruzi* DNA with maxicircle cytochrome c oxidase subunit II (COII), confirming positive samples by minicircle hypervariable DNA amplification and fragment analysis according to conditions in Table S1 in File S1 [30].

### Canine serological survey

A sample of 80 dogs (one dog per household) was screened to detect an estimated 30% seroprevalence (90% Confidence level, 0.1 precision) [31], based on the rapid tests (*Trypanosoma cruzi* Detect-Canine; Inbios, Seattle, WA, USA) sensitivity of 91% and specificity of 98% in dogs experimentally infected with *T. cruzi* [32].

Owners were asked to select an animal in the household based on the inclusion criteria: age (>6 months), health (no emaciated animals) and reproductive status (non-lactating females). Canine blood sample (2–5 mL) was obtained by venipuncture from the braquial vein and rapid tests were performed according to manufactureŕs instructions. Proportion confidence intervals were calculated without continuity correction [33].

### Qualitative data collection methods

Semi-structured interviews were conducted with key stakeholders in the region and community members: participants were selected through snowball strategies [34]. Interviews were conducted until data saturation [20], [35] was reached-no new data emerged. In addition, three communities from Comapa were selected for participatory group meetings [36] based on infestation level, social organization, socioeconomic and political dynamics. A cyclical process of joint learning, reflection, and exploration occurred during the five sessions with each community [36]. Guided discussions included food production systems, management of peridomestic animals, socioeconomic and land tenure systems, triatomine and Chagas disease knowledge and awareness, problem identification associated with Chagas disease, and community-based generation of future goals and strategies to reduce triatomine infestation.

Qualitative data were transcribed, encoded and analyzed with NVivo 9 (QSR int, Massachusetts, USA). Free nodes from patterns were created by open coding [37] and tree nodes were based on the research objectives. Pattern coding aided in the generation of categories and themes [38], [39], according to the PRECEDE-PROCEED model [40]. In addition to the emerging patterns, advanced search routines with selected keywords and nodes were run to analyze participants' perceptions regarding risk factors detected by multimodel inference.

### Identification of risk factors for house infestation with multimodel inference

Information generated in the household and entomological surveys was used to determine the relative importance (RI) of a set of variables in explaining the presence of *T. dimidiata* in domestic environments in Comapa and Zapotitlán households. The eco-bio-social variables included in the analysis were selected *a priori* based on evidence of association with infestation from published studies, known infection reservoirs, blood meal analysis and animal infection data gathered throughout the present study. Not all selected variables had significance in the univariate analysis from the KAP and entomological surveys. These variables constituted our hypothesis to explain the presence of *T. dimidiata* inside houses. Multimodel inference was used to determine the RI for each variable in the hypothesis and to explore model uncertainty for reduced models. Records (houses) with missing values were removed [41]. The resulting dataset included 449 records (houses) and 25 variables (Table S2 in File S1). Rodent survey information (*M. musculus* and *R. rattus* presence) was available for a subset of 220 houses.

To model the variable “bug presence in domestic environments” logistic regression was used with the glm function in R [42]. The Hosmer and Lemeshow test was used to evaluate the goodness of fit of the model to the data. Multicollinearity was evaluated by calculating the variance inflation factors using the vif function from the car library in R. Collinear variables were identified and dropped from the model. Multimodel inference was used to determine the relative importance of variables that best explained the presence or absence of *T. dimidiata* in the households [43]. A subset of reduced models with different combinations of variables was compared with the Akaike information criterion with a correction for finite samples (AICc) to assess model uncertainty using Akaike weights. Due to model uncertainty, weighted parameter estimates were calculated. Variable RI was estimated by adding the weights of the models with the lowest AICc in which the variables appeared.

The subset of reduced models with different variable combinations was explored using the glmulti package for R [44], [45]. Ten genetic algorithm runs (GAR) were conducted with the parameter value combinations (Methods in File S1) for the dataset that included all the houses. For each GAR the RI, weighted parameter estimates, and weighted parameter variances of all variables were obtained using the coef function in glmulti, and then averaged across all runs. Odds ratios and their confidence intervals were calculated from these average values; we interpret these intervals with caution given that they are derived from the average of weighted parameters. After the most important variables were identified with the larger dataset, 10 more GAR were conducted using these variables and the rodent information with the smaller subset of houses. The RI, weighted parameter estimates, and variance for these analyses are also reported.

## Triangulation

Methodological triangulation [34], [35] of biophysical, quantitative and qualitative social science data collection methods was used to enhance the strengths of interpretations and conclusion about risk factors. Information from interviews, document reviews, reflections, and group meeting was triangulated with information identified through surveys, multimodel inference, rodent infection and blood meal analysis. Analysis for this paper utilized a triangulation approach for converging and validating results using multiple methods. In addition, triangulation across sources [46] was utilized to understand perspectives among different interviewees.

## Results

### Biological variables associated with infestation

The baseline entomological survey revealed domestic and/or peridomestic infestation in 120 (25%) of 472 houses. Domestic infestation was detected in 101 (21%) of 472 houses. Peridomestic infestation, defined as infestation in structures not sharing a common roof with sleeping quarters, was detected in 33 (7%) of 472 houses. Infestation in both domestic and peridomestic environments was detected in 14 (3%) of 472 houses. The results of household participant searches during the follow-up survey showed that 144 (30%) of 477 houses were infested. Considering both the baseline and follow-up surveys together, colonization (i.e. nymph presence) was observed in 104 (72%) of 144 houses. Of all adults collected by the person-hour method, 38% were collected in bedrooms, 10% in chicken coops and 8% in kitchens. Interestingly, similar proportions were observed for nymphs collected in these locations (data not shown).

The self-reported presence of domestic and peridomestic animals was evaluated as a risk factor for house infestation (presence of adults, nymphs or both). The reported ownership of at least one dog showed a significant association with triatomine infestation (Table 1). Rodent surveys showed 61% infestation, with *M. musculus* and *R. rattus* as the primary rodent species. *Peromyscus mexicanus, Liomys salvini*, and *Oryzomys* spp were collected sporadically inside homes. The presence of at least one rodent per house (any species) showed a significant association with triatomine infestation (Table 1). The presence of chickens roosting or laying eggs inside the house showed a weak to moderate association (Table 1).

**Table 1:** Association of triatomine infestation according to person-hour survey and animal ownership as reported by householders, and rodent presence as recorded by rodent surveys.

Animal	No. present/Total no. (%)	Range of numbers of animals per house	Yateś chi-square	p	df	OR (95% CI)
Horse	99/467 (21)	0–4	1.20	0.273	1	
Donkey	11/467 (2)	0–2	0.73	0.395	1	
Rabbit	4/467 (1)	0–4	0.62	0.430	1	
Cow	57/467 (12)	0–12	0.63	0.429	1	
Cat	147/467 (31)	0–6	0.96	0.328	1	
Pig	62/467 (13)	0–10	0.01	0.938	1	
Dog^a^	333/466 (71)	0–9	7.61	0.006	1	2.3 (1.3–4.0)
Hen	329/467 (70)	0–100	0.26	0.600	1	
Fowl	131/467 (28)	0–50	0.80	0.373	1	
Chicks	16/467 (3)	0–41	0.33	0.566	1	
Hens laying eggs inside^b^	22/466 (5)		3.27	0.071	1	2.2 (0.9–5.5)
Hens roosting inside^c^	90/457 (20)		6.55	0.010	1	2.0 (1.2–3.3)
Rodents^d^	149/249 (61)	0–13	5.44	0.002	1	4.0 (1.1–14.1)

a Significant associations between reported ownership of animal and presence of triatomines.

b Weak association.

c Moderate association.

d Significant association between presence of any rodent species and presence of triatomines.

The biological relevance of the association between peridomestic animals and triatomine infestation was confirmed by blood meal analyses to detect DNA from humans, dogs, chickens and the two major rodent species, *R. rattus* and *M. musculus* (Table 2). Vertebrate blood was detected in 37 (18%) of 201 specimens. Most insects had fed on chickens (65%), followed by humans (51%), *R. rattus* (22%), dogs (19%) and *M. musculus* (19%). Only 12 (36%) bugs had single blood meals (6 from chickens, 5 from humans and 1 from *M. musculus*). More than half of the blood meals had two to four different blood sources. All blood meal types were identified in triatomines collected in both domestic and peridomestic environments. Three nymphs with human-mouse or human-rat blood meals were collected in sleeping quarters. A human-mouse blood meal was detected in a third instar collected in a corral located 4-8 m from domestic premises. Infection was detected in 11 (31%) of 35 triatomines with a recent vertebrate blood meal, including 33% of insects with chicken blood meals. No detectable association was identified between blood meal source and infection status (Fisheŕs exact test, p>0.5).

**Table 2:** Triatomine blood meal sources of *T. dimidiata* and *T. cruzi* infection status according to blood meal source.

Identified blood meal	No. blood meals/No. examined (%)	No. infected by PCR/No. examined (%)
Chicken	23/36 (64)	8/21 (38)
Dog	6/36 (17)	2/6 (33)
Human	18/36 (50)	6/16 (38)
*Mus musculus*	7/34 (21)	0/6 (0)
*Rattus rattus*	8/34 (24)	2/6 (33)
Vertebrate	3/36 (8)	0/3 (0)

Mixed blood meals include human, chicken, dog and mouse (2), human-chicken-dog (1), human-chicken-rat (4), chicken-dog-rat (2), human-chicken-mouse (1), human-mouse-rat (1), chicken-dog (1), chicken-mouse (1), chicken-rat (2), human-chicken (4), human-dog (1), human-rat (1) and human-mouse (1).

### *Trypanosoma cruzi* transmission indicators

*Trypanosoma cruzi* transmission was evaluated by analysis of infection in vectors, dogs and rodents (Table 3). Microscopic screening showed that 40 (31%) of 130 of the infested households had at least one infected triatomine. Infections were detected in females, males, second, third and fourth instars. Twentyeight (35%) of 80 adult dogs were seroreactive. The true prevalence adjusted for test specificity and sensitivity is estimated at 37% (25%-50%, Blakeŕs 95% Confidence Limits) (Table 3). Eleven (21%) of 53 rodents captured in homes with microscopically infected triatomines had *T. cruzi* DNA in heart tissue confirmed by COII amplification and minicircle fragment size or GPI sequence analysis. Eight (17%) of 46 *M. musculus* and three (43%) of seven *R. rattus* had *T. cruzi* infection confirmed by GPI sequence or minicircle fragment size analysis. The *T. cruzi* strain in two mice and two rats from the same household was confirmed as DTU I by sequencing GPI (Accession Nos. KJ682643-6). Eight (44%) of 18 triatomines with human blood meals had *T. cruzi* DNA as determined by minicircle fragment size.

**Table 3:** Indicators of *T. cruzi* transmission in Comapa and Zapotitlán, Jutiapa, Guatemala 2011.

Transmission indicators	No. present/Total no. (%)	95% CI
Triatomine infested households with at least one microscopically positive triatomine	40/130 (31)	23–39
Seropositive adult dogs*	28/80 (37)	25–50
PCR-positive rodents in households with infected triatomines	14/54 (26)	16–39
PCR-positive triatomines with detectable vertebrate blood meals	11/33 (33)	20–50

* True prevalence adjusted for test specificity and sensitivity (Blakeŕs 95% Confidence Limits) [61].

### Ecological variables associated with infestation

**Figure 2:**
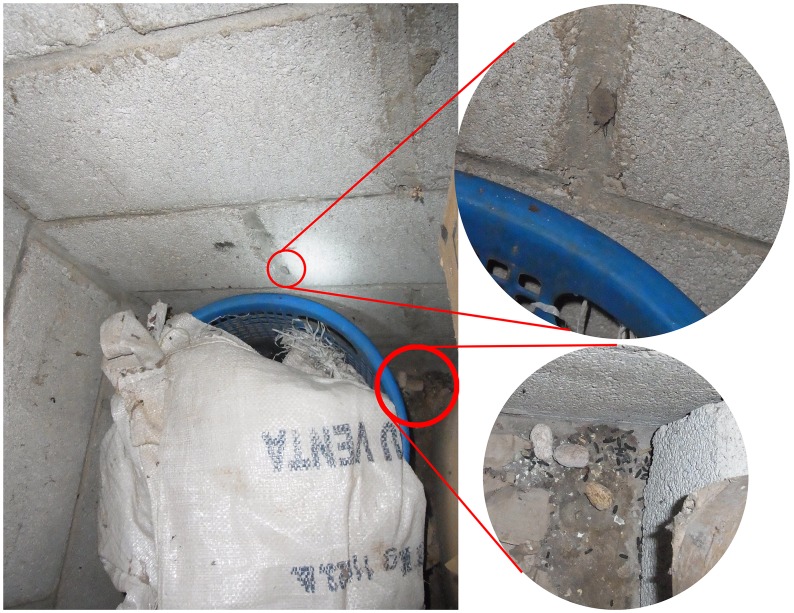
Storage facility associated with triatomine and rodent infestation. *Triatoma dimidiata* nymph found in association with a storage area with rodent feces and jocote fruit pits. Upper inset: Nymph. Lower inset: Fruit pits surrounded by rodent feces.

Reports of triatomines or bats entering or approaching the house at night were associated with infestation (Table 4). The presence in the patio of fruit-bearing trees such as avocado, coffee and jocote (*Spondias purpurea*) was found to be positively associated with triatomine presence (Table 4). Figure 2 shows a storage area in a cinder block house where nymphs were detected. As the area was investigated, rodent feces and jocote fruit pits were found on the dirt floor, suggesting a link between rodents and fruit-bearing trees in the area.

**Table 4:** Ecologic factors from the Knowledge-Attitude-Practices and entomological survey showing association with triatomine infestation, as determined by the person-hour method (baseline survey n = 472).

Variable	No. positive/Total no. (%)	Phi (*Φ*)	Odds ratio (95% CI)
Bugs enter or approach the house at night	243/469 (52)	0.215^b^	3.0 (1.9–4.9)
Bats enter or approach the house at night	307/469 (66)	0.097^a^	1.7 (1.0–2.8)
Avocado trees in the patio	230/469 (49)	0.098^a^	1.6 (1.0–2.5)
Coffee trees in the patio	307/469 (65)	0.119^b^	1.9 (1.2–3.2)
Jocote trees in the patio	346/469 (74)	0.100^a^	1.8 (1.1–3.2)

a Weak association.

b Moderate association.

### Socioeconomic variables associated with infestation

Table 5 and Table S3 in File S1 present all variables from the KAP survey and observations showing an association between socioeconomic variables and household construction with triatomine infestation. As an indication of previous vector control activity in the region, the presence of a Ministry of Health Household Vector Card in the house was found in 187 (41%) of 454 surveyed households (Table S3 in File S1). This card is placed by the vector control personnel inside houses that are visited for surveillance or insecticide applications. Households having this card had 1.7 (1.1–2.7, 95% CI) higher odds of infestation than those without it. Only 241 (53%) of 454 surveyed had heard about Chagas disease, with 52 (22%) of these individuals having received information from the vector control personnel and 94 (39%) from the Health Center. The odds of infestation were 0.4 (0.2–0.8) lower in households where someone had received information from the Health Center, and 2.5 (1.2–5.0) higher when they received information from vector control personnel. Of those who had heard about the disease, 132 (54%) of 245 reported using insecticides to control it, showing a weak positive association with infestation (phi = 0.106).

**Table 5:** Socioeconomic factors from the Knowledge-Attitude-Practices and entomological survey showing associations with triatomine infestation, as determined by the person-hour method (baseline survey, n = 472).

Variable	No. positive/Total no. (%)	Cramer's *V*	Phi (*Φ*)	Odds ratio (95% CI)
Bajareque (mud and stick) walls present	214/468 (46)		0.134^b^	1.9 (1.2–3.9)
Plastering condition (partial, complete, absent)		0.139^b^		
Cinder block walls	122/468 (26)		−0.146^b^	0.4 (0.2–0.7)
Tile roofs	76/468 (16)		0.107^a^	1.9 (1.1–3.3)
Cement tile floors	94/468 (20)		−0.146^b^	0.3 (0.2–0.7)
Earthen floors	309/468 (66)		0.201^b^	3.4 (1.9–6.0)
Cement slab floors	137/468 (29)		−0.121^b^	0.5 (0.3–0.8)
Eaves	378/447 (85)		0.110^b^	2.5 (1.1–5.3)
Socioeconomic index 1		0.118^b^		
Socioeconomic index 2		0.222^b^		

a Weak association.

b Moderate association.

Socioeconomic index 1: variables for having electricity at home, owning a cellular phone, using electricity for cooking, and using electricity for lighting the house at night, Socioeconomic index 2: variables for having electricity at home; owning a cellular phone; using electricity for cooking; using electricity for lighting the house at night; and number of chickens, cows, and pigs owned. Table S2 in File S1 shows the variables associated with infestation that were used to derive Socioeconomic index 1 and 2.

Only 26% of houses were constructed in part from cinder blocks; most houses were built of adobe (45%) and bajareque (mud with sticks and sometimes wood) or mud (46%). Sixteen percent of households still had a tile roof. In addition, 66% of houses had dirt floors. For ventilation purposes, eaves (open space between the roof and the wall), another house construction feature in the area, were present in 85% of houses. Household partial wall plastering was positively associated with infestation. Cement or tile floors were protective (OR = 0.3, 95% CI 0.2–0.7) whereas tile roofs increased infestation risk (OR = 1.9, 95% CI 1.1–3.3). Cinder block walls were protective. Walls containing bajareque, but not adobe, were a risk factor (OR = 1.9, 95% CI 1.2–3.9). There was a moderate positive association between socioeconomic indices and the presence of triatomines. Firewood was used for cooking by 98% of households. The house was often used as a storage facility: 72% stored grains in metal containers, 41% stored grains in bags, and 20% stored firewood.

### Identification of risk factors for house infestation

Logistic regression analysis was used to model the variable “bug presence in domestic environments” (Hosmer-Lemeshow fit test, chi-squared = 4.20, p = 0.84) for the first dataset including all the houses. The variables “total number of humans in the house” and “type of wall plaster (exterior)” were dropped due to collinearity, but the variables “number of persons per room” and “type of wall plaster (interior)” were retained. Implementation of the glmulti genetic algorithm made it possible to fit a subset of reduced logistic models with different variable combinations, which allowed to estimate the relative importance of the variables in the hypothesis and to assess model uncertainty. The average number of generations per run was 1,653±726 (100 models fitted per generation) before convergence. The exploration of multiple models with the genetic algorithm revealed that there was high uncertainty and that there was no single best model among all the models compared in the 10 GAR. The reduced model with the lowest AICc in the subset of models explored was “bugsInside∼Intercept + CoffeeTrees + RoofType + FloorType + InternalPlaster + CellPhone + Dogs”, which had an Akaike weight of 0.26 and an AICc of 433.26 compared to an AICc of 468.32 in the full model (24 variables). An Akaike weight under 0.90 indicates that there is not a single reduced model that could be considered the best model ( = hypothesis) among those compared. Therefore a model using weighted parameters like the one presented here is recommended. After obtaining the average across all runs, we found that the most important variables (RI≥0.75, Table 6), among those considered to explain the presence of bugs in houses in this region of Jutiapa were the type of internal wall plastering (RI = 0.98), type of floor (RI = 0.94) and number of dogs (RI = 0.98) (Table 6). The presence of coffee trees around the house and the type of roof also had high RI values (RI = 0.85 and 0.83, respectively). All other variables had an RI under less than 0.75 (Table S4 in File S1).

**Table 6:** Model results showing the most important individual factors that increase (or decrease) the relative odds of *Triatoma dimidiata* presence inside households, keeping all other conditions constant.

Factors (odds_1_/odds_2_)	Average relative importance (RI)	Average weighted estimate	Average weighted variance	Odds ratio (95% CI)
Intercept	1	−2.48	0.52	
1–2 dogs/0 dogs	0.99	0.74	0.12	2.09 (1.06–4.13)
>2 dogs/0 dogs	0.98	1.08	0.16	2.95 (1.36–6.40)
Partial interior plaster/complete interior plaster	0.98	1.44	0.32	4.20 (1.37–12.83)
No interior plaster/complete interior plaster	0.98	0.34	0.19	1.40 (0.59–3.33)
Non-dirt floor/dirt floor	0.94	−0.82	0.12	0.44 (0.22–0.87)
Coffee trees yes/no	0.85	0.50	0.11	1.64 (0.85–3.16)
Clay tile or plant material roof/metal-sheet roof	0.83	0.51	0.12	1.67 (0.84–3.29)

The table shows only variables with RI>0.80.

Of these variables, the ones that appeared to increase the relative odds of bug presence and detection, keeping all other conditions constant, were (a) partial or no plastering in the house, in comparison to houses with complete plastering; (b) having at least one dog; (c) having coffee trees around the house; and (d) having a roof made of materials other than metal-sheet like clay tiles or plant leaves. On the other hand, cement or tile floors reduced the odds of bug presence inside the house in comparison to dirt floors.

A logistic regression model using only the most important variables identified previously (type of roof, type of floor, type of interior plastering, number of dogs and presence of coffee trees), and the variables altitude, *M. musculus* presence and *R. rattus* presence was fitted to a subset of data with rodent information. This model showed a good fit to explain the infestation data (Hosmer-Lemeshowfit test, chi-squared = 5.32, p = 0.72). The exploration of multiple reduced models with the genetic algorithm (with an average 214±28 generations per run) again revealed high uncertainty and there was no single best model among the models compared in the 10 GAR. The model with the lowest AICc “BugsInside ∼ Intercept + RoofType + FloorType+ Dogs + *Mus musculus* presence”, with an Akaike weight of 0.05 and an AICc of 212.38 compared to an AICc of 217.92 in the full model. Using multimodel inference it was determined that the most important variables (RI≥0.75) among those considered to explain the presence of bugs in this subset of houses were *M. musculus* presence (RI = 0.89, OR = 2.13, 95% CI 1.93–4.89) and type of roof (RI = 0.77, OR = 2.02, 95% CI 0.71–5.77). All other variables had an RI less than 0.75 for this subset of data.

### Perceptions and practices related to chickens, rodents, construction and environment as risk factors

Several study participants mentioned having seen triatomines more often around chickens and chicken coops, especially at night. Qualitative data from participant group meetings indicated that hens and chickens were left inside the house at night to roost and during the day to lay eggs; this seemed to be an established practice in the study area. According to the following quote from a group session, one participant recognized that having chickens inside the house represented a risk for triatomine infestation.

“I have proved that chickens do in fact attract the bugs, because previously we left them all inside, a while ago. Where they slept, there were the bugs. Then my husband decided to take them outside, in a coop outside” (*Group meeting 9.1, 2011*).

In addition, vector control personnel recognize chickens laying eggs under the bed as a risk factor.

“People continue to have chickens lay their eggs underneath the beds and, unfortunately due to extreme poverty, the walls have all the conditions for the bug to keep reproducing inside the houses” (Interview 1, February 25, 2011).

The majority of participants (68%) were concerned about rodents transmitting diseases. Interestingly, 61% of the households reported having few mice and 50% few rats. Nevertheless, 53% perceived that there were many mice in the community and 56% that there were many rats.

Several participants emphasized the influence of house construction materials and styles on the presence of triatomines and rodents in the household. Innovations mentioned by participants to prevent house infestation by triatomines and rodents include refurbishing walls, placing new roof tiles, building a cement floor and closing eaves.

“The interviewee mentioned he had refurbished the walls himself. He also mentioned having sealed the eaves, to prevent bugs and rats, and in fact the wall (mud renovated) reached the ceiling (sheet). He even mentioned that before renovating the house walls and eaves he had problems with animals, but not after that.” (Reflective journal EP, October 12, 2012).

“More triatomines could be found inside the houses when we had tile roofs” (Group meeting, November, 2012).

Traditional house construction styles in the area have been considered risk factors for the presence of triatomines by vector control personnel.

“…Usually we find in these families all the factors that contribute to triatomine presence, such as a (dirt) floor, bajareque or mud walls –they make own adobe –, thatched roofs, and they share their habitat with pets: dogs, pigs, hens […]. They are people with limited resources […]. They cook with firewood, and store firewood around the house” (Interview I.11, June 17, 2011).

Finally, several study participants believed leaf litter to be associated with bug infestation. “Having leaf litter around the house makes having bugs (triatomines) there more likely” (Reflective journal EP from group meeting 2.3, November 12, 2012).

## Discussion

The factors that were found to play a role in persistent intradomiciliary triatomine infestation in this region of Guatemala include the presence of parasite reservoir hosts such as dogs and rodents, the presence of chickens as blood sources, house construction methods, and environmental conditions around the household that are related to cultural practices and socioeconomic conditions. We developed a multilevel triangulation mixed methods design that combines biostatistical, biological and social science methods to improve our understanding of the eco-epidemiology of Chagas disease at the local level, and that provides evidence for IVM.

Our study showed that house construction methods and materials related to local cultural and socioeconomic conditions are associated with infestation. Construction materials such as bajareque (mud and sticks) and tile roofs are associated with infestation, whereas cement and brick floors are protective. The association of construction materials and triatomines is not homogeneous. A study in a nearby municipality identified primarily adobe walls as a risk factor [12] whereas tile roofs were found to be a protective factor at the department level [28]. In agreement with our study, King *et al* found dirt floors to be a risk factor in Jutiapa but not in a northern department of the country [28]. The presence of different *T. dimidiata* phylogenetic groups across the endemic area from Mexico to Ecuador suggests that different vector populations may be associated with different ecotopes, depending on environmental conditions [47]. These local differences indicate that eco-bio-social conditions vary across regions, and generalizations regarding house construction materials as risk factors may not be possible for *T. dimidiata*.

In our study, partial wall plastering was a risk factor when compared to complete wall plastering. On the other hand, the lack of wall plastering was associated with infestation in a different region of Guatemala [48]. Improving the quality of plastering has been suggested as a control method for triatomines in Central America [49]. Our results indicate that house improvement methods will need to take into account local socioeconomic conditions and practices related to construction, which will vary across regions.

The role of vegetation around the household needs more analysis. The type of vegetation (i.e. cropland and grassland) was previously shown to be related to *T. dimidiata* infestation in different regions of Guatemala [28]. In our study, the presence of coffee trees appears to be associated with triatomine infestation. Whether this is related to the availability of host nesting sites close to the house or other environmental conditions remains to be determined. Householders plant coffee, avocado and jocote (local fruit) trees in their patios for personal consumption. These plants produce fruits and leaf litter, providing ideal breeding sites and food for reservoir hosts such as rats in and around the house [50]. Interestingly, *R. rattus* is commonly called the “tile roof rat” because of its arboreal nesting habits. The importance of tile roofs, coffee trees and rodent infestations as shown in the model may be related to the presence of domestic and peridomestic rodent nests that act as links with the domestic transmission cycle.

Practices regarding animals that pose a risk factor for infestation and transmission may also vary according to local perceptions and needs. Blood meal analyses showed that chickens, rodents and dogs are important blood sources in this region, as in another study in a nearby Municipality [51]. Chickens are an important part of household nutrition and economy, and are allowed to nest inside the house. The practice of having chickens laying eggs in the house showed a weak association with infestation and was identified by participants as a risk factor that could be reduced by moving the animals outside of the house. The importance of chickens nesting inside the house was shown for *T. infestans* [18], whereas keeping chickens in coops was a risk factor for *T. dimidiata* in Mexico [3]. Similar to an earlier study in Mexico, we found that the presence of more than two dogs per household was an important risk factor, suggesting that animal densities are a determinant of infestation [3]. The bug mortality during transport could lead to bias in the relative frequencies of the different blood meals which could change the relative importance of these animals in maintaining triatomine colonies. However, we were able to triangulate the identification of chicken, dog, mouse and rat blood meals with the evidence of infection in dogs, mice and rats and the perceptions regarding rodents and chickens as associated with infestation. These data taken together strengthen the inclusion of these animals in the model. The use of species-specific PCR limits the identification of other potential important blood sources to be included in the model, such as cats. Moreover, several *cyt b* positive samples did not disclose any specific blood source. Future studies to sequence the universal *cyt b* gene should provide additional insight into other potential habitats for triatomines and the role of other mammals in transmission in and around the house.

This study confirms *T. cruzi* transmission risk in this region. The high proportion of infected triatomines having human blood meals, together with the evidence of previous infection in dogs and *T. cruzi* DTU I in rodents, suggests a high risk for infection in domestic environments. The association between rodent and triatomine infestation; the presence of rodent blood meals and the presence of infected rodents in households with infected triatomines indicate that rodents are an important reservoir in the study area.

Although synanthropic rodents were previously found to be infected with *T. cruzi* [52], [53], their role in domestic transmission has not been fully documented. The molecular detection of a blood meal indicates blood meals taken within the last 2–3 weeks [53]. The detection of multiple blood meals in nymphs, including human blood meals in outdoor structures, suggests that immature stages actively move in search of blood meals, as found in another Municipality [51] and for *T. infestans* in Peru and Argentina [4], [6]. This may be a situation that facilitates house re-infestation, as nymphs may find peridomestic shelters in rodent nesting areas, found within and outside the household, that do not receive insecticide treatment. A participant perceived construction materials as risk factors for triatomines and rodent infestation. Also, leaf litter found outside the household was noted as associated with triatomines. It is tempting to speculate that these factors represent an ecological niche for rodent nests that link peridomestic and domestic transmission cycles. Control methods such as environmental management to reduce rodent habitats could be developed to target nesting sites, as recently suggested for *Triatoma brasiliensis* [54].

The variables included in the multivariate model represent our hypothesis of the factors that are associated with (or that could be the cause of) house infestation by *T. dimidiata* in the region, as a proxy to transmission risk to humans. Our modeling technique (multimodel inference) allowed us to estimate the relative importance of the variables in our hypothesis. We were also able to explore a subset of reduced models with different combinations of the variables in the hypothesis in order to detect model uncertainty: given the high uncertainty found, a model with weighted parameters is recommended for this data. One limitation of our model is that triatomine infestation was detected by the person-hour method. This method is known to have a low sensitivity, especially at low vector densities and in complex peridomestic environments [54], [55]. Including reports by householders could improve infestation detection [55]. However, the current study focused on identifying risk factors for intradomiciliary infestation and collections by participants did not include capture site information. Future studies of this species in Guatemala could include repeated sampling to estimate detection error for distinct ecotopes [54].

A second limitation of the model is that it is dependent on the sample size and the data obtained. Some of the factors we hypothesized to be associated with infestation fell below the 20% prevalence level (e.g., the prevalence of chickens entering the house to lay eggs was 5%), and had to be aggregated to create a new factor (“presence of animals inside the house”); as a result they could not be tested individually. Additionally, some of the factors included in the model (i.e., wall plastering) are complex to measure and were secondarily derived from field observations (detailed data for the extent of plastering of each wall), which may have affected the ultimate association with infestation data. Finally, the presence of vector control cards in 41% of the households indicates a previous visit by the Ministry of Health that may include insecticide spraying or other control activities. We were not able to include in the model previous spraying activity because not all households had this information. Given that reinfestation odds vary depending on the evaluation time after spraying, the model could be improved if previous spraying information were gathered for each house [8].

The triangulation of data from multimodel inference, blood meal analysis, biological indicators of transmission and social science analyses supports the notion that rodents and dogs may be involved in triatomine infestation and in maintaining the parasite transmission cycle in the house, as has been observed for dogs, cats [56] and guinea pigs [6] in connection with *T. infestans*. Adult dog seroprevalence was in a range similar to that observed in other studies with domestic dogs in the region, ranging from 10% to 30% [57], [58]. Considering the high seroprevalence and the low number of dog blood meals from bugs collected inside the households, it is tempting to speculate that dogs may become infected orally by ingesting infected rodents or triatomines. In a natural reserve in Brazil, a similar proportion of dogs were found to be infected, showing that transmission in dogs is not circumscribed to synanthropic environments [59].

Future studies should focus on clarifying the role that rodents and dogs play in triatomine infestation and disease transmission, and may provide control strategies to reduce their impact as reservoirs. Given the high rodent infestation and evidence of infection of mice and rats with *T. cruzi* in the study area in addition to their importance in the transmission of other diseases [60], we propose that an IVM program should also include synanthropic rodent control to reduce *T. cruzi* transmission.

## Supporting Information

File S1 **Supplementary material including Tables S1, S2, S3 and S4.**
(DOC)

## References

[1] WHO (2007) WHO Report of the global partners' meeting on neglected tropical diseases 2007- a turning point. Geneva, Switzerland: World Health Organization. 75 p.

[2] Sommerfeld J, Kroeger A (2012) Eco-bio-social research on dengue in Asia: a multicountry study on ecosystem and community-based approaches for the control of dengue vectors in urban and peri-urban Asia. Pathog Glob Health 106: 428–435. 23318234 10.1179/2047773212Y.0000000055 PMC3541880

[3] Dumonteil E, Nouvellet P, Rosecrans K, Ramirez-Sierra MJ, Gamboa-Leon R, et al (2013) Eco-Bio-Social determinants for house infestation by non-domiciliated *Triatoma dimidiata* in the Yucatan Peninsula, Mexico. PLoS Negl Trop Dis 7: e2466. 24086790 10.1371/journal.pntd.0002466 PMC3784500

[4] Vazquez-Prokopec GM, Spillmann C, Zaidenberg M, Gurtler RE, Kitron U (2012) Spatial heterogeneity and risk maps of community infestation by *Triatoma infestans* in rural northwestern Argentina. PLoS Negl Trop Dis 6: e1788. 22905276 10.1371/journal.pntd.0001788 PMC3419179

[5] Hashimoto K, Alvarez H, Nakagawa J, Juarez J, Monroy C, et al (2012) Vector control intervention towards interruption of transmission of Chagas disease by *Rhodnius prolixus*, main vector in Guatemala. Mem Inst Oswaldo Cruz 107: 877–887. 23147143 10.1590/s0074-02762012000700007

[6] Levy MZ, Quispe-Machaca VR, Ylla-Velasquez JL, Waller LA, Richards JM, et al (2008) Impregnated netting slows infestation by *Triatoma infestans* . Am J Trop Med Hyg 79: 528–534. 18840739 PMC2659296

[7] Ceballos LA, Piccinali RV, Marcet PL, Vazquez-Prokopec GM, Cardinal MV, et al (2011) Hidden sylvatic foci of the main vector of Chagas disease *Triatoma infestans*: threats to the vector elimination campaign? PLoS Negl Trop Dis 5: e1365. 22039559 10.1371/journal.pntd.0001365 PMC3201917

[8] Manne J, Nakagawa J, Yamagata Y, Goehler A, Brownstein JS, et al (2012) Triatomine infestation in Guatemala: spatial assessment after two rounds of vector control. Am J Trop Med Hyg 86: 446–454. 22403315 10.4269/ajtmh.2012.11-0052 PMC3284360

[9] Zeledon R, Montenegro VM, Zeledon O (2001) Evidence of colonization of man-made ecotopes by *Triatoma dimidiata* (Latreille, 1811) in Costa Rica. Mem Inst Oswaldo Cruz 96: 659–660. 11500765 10.1590/s0074-02762001000500012

[10] Aiga H, Sasagawa E, Hashimoto K, Nakamura J, Zúniga C, et al (2012) Chagas disease: Assessing the existence of a threshold for bug infestation rate. Am J Trop Med Hyg 86: 972–979. 22665603 10.4269/ajtmh.2012.11-0652 PMC3366542

[11] Hashimoto K, Cordon-Rosales C, Trampe R, Kawabata M (2006) Impact of single and multiple residual sprayings of pyrethroid insecticides against *Triatoma dimidiata* (Reduviiade; Triatominae), the principal vector of Chagas disease in Jutiapa, Guatemala. Am J Trop Med Hyg 75: 226–230. 16896123

[12] Bustamante DM, Monroy C, Pineda S, Rodas A, Castro X, et al (2009) Risk factors for intradomiciliary infestation by the Chagas disease vector *Triatoma dimidiata* in Jutiapa, Guatemala. Cad Saude Publica 25 Suppl 1S83–92. 19287870 10.1590/s0102-311x2009001300008

[13] Gurevitz JM, Ceballos LA, Gaspe MS, Alvarado-Otegui JA, Enriquez GF, et al (2011) Factors affecting infestation by *Triatoma infestans* in a rural area of the humid Chaco in Argentina: a multi-model inference approach. PLoS Negl Trop Dis 5: e1349. 22028941 10.1371/journal.pntd.0001349 PMC3196485

[14] Zeledon R, Solano G, Swartzwelder JC (1970) Sources of blood for *Triatoma dimidiata* (Hemiptera: Reduviidae) in an endemic area of Chagas' disease in Costa Rica. J Parasitol 56: 102. 4984080

[15] Gurtler RE, Ceballos LA, Ordonez-Krasnowski P, Lanati LA, Stariolo R, et al (2009) Strong host-feeding preferences of the vector *Triatoma infestans* modified by vector density: implications for the epidemiology of Chagas disease. PLoS Negl Trop Dis 3: e447. 19478849 10.1371/journal.pntd.0000447 PMC2682203

[16] Grijalva MJ, Palomeque FS, Villacis AG, Black CL, Arcos-Teran L (2010) Absence of domestic triatomine colonies in an area of the coastal region of Ecuador where Chagas disease is endemic. Mem Inst Oswaldo Cruz 105: 677–681. 20835616 10.1590/s0074-02762010000500013

[17] Schwarz A, Helling S, Collin N, Teixeira CR, Medrano-Mercado N, et al (2009) Immunogenic salivary proteins of *Triatoma infestans*: development of a recombinant antigen for the detection of low-level infestation of triatomines. PLoS Negl Trop Dis 3: e532. 19841746 10.1371/journal.pntd.0000532 PMC2760138

[18] Cecere MC, Gurtler RE, Chuit R, Cohen JE (1997) Effects of chickens on the prevalence of infestation and population density of *Triatoma infestans* in rural houses of north-west Argentina. Med Vet Entomol 11: 383–388. 9430119 10.1111/j.1365-2915.1997.tb00426.x

[19] Beteta H, Castro M, Rodríguez E, Benedicto Estrada S, Anzueto M, et al.. (2006) Mapas de pobreza en Guatemala al 2002. Guatemala: Gobierno de Guatemala, SEGEPLAN. 1–47 p.

[20] Creswell J, Plano Clark V (2007) Designing and conducting mixed methods research. Thousand Oaks, CA.: Sage Publications, Inc. 275 p.

[21] Scheaffer RL, Mendenhall III W, Ott RL, Gerow KG (2012) Elementary survey sampling. Boston, MA: Brooks/Cole. 436 p.

[22] Bennett S, Woods T, Liyanage WM, Smith DL (1991) Simplified general method for cluster-sample surveys of health in developing countries. Rapport trimestriel de statistiques sanitaires mondiales 44: 98–106. 1949887

[23] World Health Organization (2005) Immunization coverage cluster survey: Reference manual. Geneva, Switzerland: WHO Document Production Services. 115 p.

[24] Demidenko E (2007) Sample size determination for logistic regression revisited. Stat Med 26: 3385–3397. 17149799 10.1002/sim.2771

[25] Demidenko E (2008) Sample size and optimal design for logistic regression with binary interaction. Stat Med 27: 36–46. 17634969 10.1002/sim.2980

[26] WHO (2008) Advocacy, communication and social mobilization for TB control: A guide to developing knowledge, attitude and practice surveys. Geneva. Switzerland: World Health Organization. 60 p.

[27] Dillman DA, Smyth JD, Melani L (2009) Internet, mail, and mixed-mode surveys: The tailored design method. Hoboken, NJ: John Wiley & Sons, Inc. 512 p.

[28] King RJ, Cordon-Rosales C, Cox J, Davies CR, Kitron UD (2011) *Triatoma dimidiata* infestation in Chagas disease endemic regions of Guatemala: comparison of random and targeted cross-sectional surveys. PLoS Negl Trop Dis 5: e1035. 21532742 10.1371/journal.pntd.0001035 PMC3075228

[29] Healey J (2012) Statistics: A tool for social research. Belmont, CA: Wadsworth Cengage Learning. 512 p.

[30] Messenger LA, Llewellyn MS, Bhattacharyya T, Franzen O, Lewis MD, et al (2012) Multiple mitochondrial introgression events and heteroplasmy in *Trypanosoma cruzi* revealed by maxicircle MLST and next generation sequencing. PLoS Negl Trop Dis 6: e1584. 22506081 10.1371/journal.pntd.0001584 PMC3323513

[31] Malhotra RK, Indrayan A (2010) A simple nomogram for sample size for estimating sensitivity and specificity of medical tests. Indian J Ophthalmol 58: 519–522. 20952837 10.4103/0301-4738.71699 PMC2993983

[32] Rosypal AC, Hill R, Lewis S, Barr SC, Valadas S, et al (2011) Evaluation of a rapid immunochromatographic dipstick test for detection of antibodies to *Trypanosoma cruzi* in dogs experimentally infected with isolates obtained from opossums (*Didelphis virginiana*), armadillos (*Dasypus novemcinctus*), and dogs (*Canis familiaris*) from the United States. J Parasitol 97: 140–143. 21348621 10.1645/GE-2559.1

[33] Newcombe RG (1998) Two-sided confidence intervals for the single proportion: Comparison of seven methods. Stat Med 17: 857–872. 9595616 10.1002/(sici)1097-0258(19980430)17:8<857::aid-sim777>3.0.co;2-e

[34] Patton M (2002) Qualitative research & evaluation methods Thousand Oaks, CA: Sage Publications. A2 p.

[35] Padgett DK (2012) Qualitative and mixed methods in public health. Thousand Oaks, CA.: Sage Publications, Inc. 285 p.

[36] Chevalier J, Buckles D (2013) Participatory action research: Theory and methods for engaged inquiry. New York, NY: Routledge. pp. 496.

[37] Strauss A, Corbin J (1998) Basics of Qualitative Research, techniques and procedures for developing grounded theory. Thousand Oaks, CA: Sage Publications. 312 p.

[38] Stake RE (2010) Qualitative research: Studying how things work. New York, NY: The Guilford Press. 243 p.

[39] Gibbs GR (2007) Analyzing qualitative data. Thousand Oaks, CA: SAGE Publications. 160 p.

[40] Green LW, Kreuter MW (2005) Health program planning: An educational and ecological approach. New York, NY: McGraw-Hill. 621 p.

[41] Zuur AF, Ieno E, Walker N, Saveliev A, Smith G (2009) Mixed effects models and extensions in ecology with R. New York: Springer. 574 p.

[42] Team R (2013) R: A language and environment for statistical computing. In: Computing RFfS, editor. Vienna, Austria. Available: http://www.R-project.org/ Accessed 2014 Mar 10.

[43] Burnham K, Anderson D (2002) Model selection and multimodel inference. New York: Springer. 488 p.

[44] Calcagno V, Mazancourt C (2010) glmulti: an R package for easy automated model selection with (generalized) linear models. J Stat Softw 34: 1–29.

[45] Calcagno V (2013) glmulti: Model selection and multimodel inference made easy. R package version 1.0.7. Available: http://cran.r-project.org/web/packages/glmulti/index.html Accessed 2014 Mar 10.

[46] Erlandson D, Harris E, Skipper B, Allen S (1993) Doing naturalistic inquiry: A guide to methods. Newbury Park, CA.: Sage Publications. 198 p.

[47] Monteiro FA, Peretolchina T, Lazoski C, Harris K, Dotson EM, et al (2013) Phylogeographic pattern and extensive mitochondrial DNA divergence disclose a species complex within the Chagas disease vector *Triatoma dimidiata* . PLoS One 8: e70974. 23940678 10.1371/journal.pone.0070974 PMC3733668

[48] Weeks EN, Cordon-Rosales C, Davies C, Gezan S, Yeo M, et al. (2013) Risk factors for domestic infestation by the Chagas disease vector, *Triatoma dimidiata* in Chiquimula, Guatemala. Bull Entomol Res: 1–10. 10.1017/S000748531300014X 23597014

[49] Lucero DE, Morrissey LA, Rizzo DM, Rodas A, Garnica R, et al (2013) Ecohealth interventions limit triatomine reinfestation following insecticide spraying in La Brea, Guatemala. Am J Trop Med Hyg 88: 630–637. 23382173 10.4269/ajtmh.12-0448 PMC3617845

[50] Clark D (1982) Foraging behavior of a vertebrate omnivore (*Rattus rattus*): Meal structure, sampling, and diet breadth. Ecology 63: 763–772.

[51] Pellecer MJ, Dorn PL, Bustamante DM, Rodas A, Monroy MC (2013) Vector blood meals are an early indicator of the effectiveness of the Ecohealth approach in halting Chagas transmission in Guatemala. Am J Trop Med Hyg 88: 638–644. 23382165 10.4269/ajtmh.12-0458 PMC3617846

[52] Zeledon R, Solano G, Burstin L, Swartzwelder JC (1975) Epidemiological pattern of Chagas' disease in an endemic area of Costa Rica. Am J Trop Med Hyg 24: 214–225. 804266 10.4269/ajtmh.1975.24.214

[53] Pinto J, Roellig DM, Gilman RH, Calderon M, Bartra C, et al (2012) Temporal differences in blood meal detection from the midguts of *Triatoma infestans* . Rev Inst Med Trop Sao Paulo 54: 83–87. 22499421 10.1590/s0036-46652012000200005

[54] Valenca-Barbosa C, Lima MM, Sarquis O, Bezerra CM, Abad-Franch F (2014) Modeling disease vector occurrence when detection is imperfect II: Drivers of site-occupancy by synanthropic *Triatoma brasiliensis* in the Brazilian northeast. PLoS Negl Trop Dis 8: e2861. 24811125 10.1371/journal.pntd.0002861 PMC4014420

[55] Abad-Franch F, Vega M, Rolón M, Santos W, Rojas de Arias A (2011) Community participation in Chagas disease vector surveillance: Systematic review. PLoS Negl Trop Dis 5: e1207. 21713022 10.1371/journal.pntd.0001207 PMC3119642

[56] Cardinal MV, Lauricella MA, Marcet PL, Orozco MM, Kitron U, et al (2007) Impact of community-based vector control on house infestation and *Trypanosoma cruzi* infection in *Triatoma infestans*, dogs and cats in the Argentine Chaco. Acta Trop 103: 201–211. 17686448 10.1016/j.actatropica.2007.06.007 PMC2931801

[57] Pineda V, Saldana A, Monfante I, Santamaria A, Gottdenker NL, et al (2011) Prevalence of trypanosome infections in dogs from Chagas disease endemic regions in Panama, Central America. Vet Parasitol 178: 360–363. 21273002 10.1016/j.vetpar.2010.12.043

[58] Montenegro VM, Jimenez M, Dias JC, Zeledon R (2002) Chagas disease in dogs from endemic areas of Costa Rica. Mem Inst Oswaldo Cruz 97: 491–494. 12118277 10.1590/s0074-02762002000400006

[59] Rocha FL, Roque AL, de Lima JS, Cheida CC, Lemos FG, et al (2013) *Trypanosoma cruzi* infection in neotropical wild carnivores (Mammalia: Carnivora): at the top of the *T. cruzi* transmission chain. PLoS One 8: e67463. 23861767 10.1371/journal.pone.0067463 PMC3701642

[60] Davis S, Calvet E, Leirs H (2005) Fluctuating rodent populations and risk to humans from rodent-borne zoonoses. Vector Borne Zoonotic Dis 5: 305–314. 16417426 10.1089/vbz.2005.5.305

[61] Reiczigel J, Földi J, Ózsvári L (2010) Exact confidence limits for prevalence of a disease with an imperfect diagnostic test. Epidemiol Infect 138: 1674–1678. 20196903 10.1017/S0950268810000385

