# Supplementary material for: Ecological, Social and Biological Risk Factors for Continued Trypanosoma cruzi Transmission by Triatoma dimidiata in Guatemala
Source: PLoS One. 2014 Aug 29;9(8):e104599. doi: 10.1371/journal.pone.0104599 (PMC4149347; doi:10.1371/journal.pone.0104599)
Supplement: File S1 — Supplementary material including Tables S1, S2, S3 and S4. (DOC) [file pone.0104599.s001.doc]

**Supplementary material**

**Methods.**

Table S1. Polymerase chain reaction conditions for blood meal analysis and *T. cruzi* amplification.

| Molecular target (size in bp) | Forward Primer 5´-3´ (name) | Reverse Primer 5´-3´ (name) | Reference |
| --- | --- | --- | --- |
| Universal vertebrate cyt*b* | CCA TCC AAC ATY TCA DCA TGA TGA AA (cytB1-F) | GCH CCT CAG AAT GAT ATT TGK CCT CA (cytB2-R) |  |
| Human β-globin | CAA CTT CAT CCA CGT TCA CC (PC04) | GAA GAG CCA AGG ACA GGT AC (Gh20) |  |
| Chicken ^a^ | GAAGAGGATAAGTAGGATGGTGAAG (Chick1123R) | TAACCTGAATCGGAAGCCAACC (UNFOR1029) |  |
| Dog ^a^ | GGAATTGTACTATTATTCGCAACCAT (Dog368F) | GGTTGTCCTCCAATTCATGTTA (UNREV1025) |  |
| *Rattus rattus* cyt*b* (104) ^b^ | TT CCT CCA TGT AGG CCG AGG G (RrCytb_F) | TGA ATG CGG TTG CTA TGA CTG C (RrCytb_R) | This study |
| *Mus musculus* cyt*b* (151) ^b^ | TAC ACG CAA ACG GAG CCT CAA (MmCytb_F) | ACG TAG CCT ATA AAT GCT GTG GCT (MmCytb_R) | This study |
| *T. cruzi* minicircle^c^ | AAA TAA TGT ACG GG(T/G) GAG ATG CAT GA (S35) | GGG TTC GAT TGG GGT TGG TGT (S36) |  |
| *T. cruzi* cyt oxidase II^d^ | GTT ATT ATC TTT TGT TTG TTT TGT GTG (COII Fwd) | AAC AAT TGG CAT AAA TCC ATG T (COII Rvs) |  |
| *T. cruzi*  GPI^e^ | GGC ATG TGA AGC TTT GAG GCC TTT TTC AG (GPI_For) | TGT AAG GGC CCA GTG AGA GCG TTC GTT GAA (GPI_Rev) |  |
| *T. cruzi*  GPI^e^ | TGG TCG ACT TGG AAT CAA CTC T (GPI_INT_F2) | CTG AAG TTG TRG AAG AAT GGA C (GPI_INT_R2) |  |

^a^ Chicken and dog reaction conditions: 0.04 mM each dNTP, 1.5 mM MgCl_2_, 0.1 μM each primer, 0.02 U/μL Taq (Promega), 2 μL DNA in 50 μL. 95°C, 5 min, 29 cycles at 95°C 1 min, 58°C 1 min, 72°C, 1 min, extension 7 min at 72°C. ^b^ Rodent reaction conditions: 0.2 mM e/dNTP, 1.5 mM MgCl_2_. 1.0 μM each primer, 0.5 U/μL Taq in 20 μL final volume 94°C, 4 min, 35 cycles at 94°C 30 s, 55°C 30 s, 72°C 30 s, extension 10 min at 72°C

^c^Minicircle: 0.4 μM each primer, 1X Master Mix GoTaq Hot Start (Promega), 3 μL DNA in 25 μL. 94°C, 5 min, 30 cycles at 94°C 30 sec, 64°C 30 sec, 72°C, 50 sec, final extension 7 min at 72°C.

^d^COII: 0.4 μM each primer, 1X Master Mix GoTaq Hot Start (Promega), 1 μL DNA in 25 μL. 94°C, 3 min, 15 cycles at 94°C 30 sec, 65°C 30 sec (touchdown 1°C/sec), 72°C, 30 sec followed by 25 cycles at 94°C 30 sec, 50°C 30 sec, 72°C, 30 sec, final extension 10 min at 72°C.

^e^GPI External PCR: 0.5 μM each primer, 1X Master Mix GoTaq Hot Start (Promega), 1 μL DNA in 25 μL. 95°C, 3 min, 30 cycles at 95°C 30 sec, 60°C 45 sec (touchdown 1°C/sec), 72°C 90 sec, final extension 5 min at 72°C

GPI Internal PCR: 0.5 μM each primer, 1X Master Mix GoTaq Hot Start (Promega), 1 μL DNA external PCR in 25 μL. 95°C, 3 min, 15 cycles at 95°C 30 sec, 70°C 30 sec (touchdown 1°C/sec), 72°C 30 sec, followed by 15 cycles at 95°C 30 sec, 55°C 30 sec, 72°C, 30 sec, final extension 5 min at 72°C.

**Identification of risk factors for house infestation using multimodel inference: explanatory variables**

With 24 explanatory variables it is possible to fit 2^24^ different models (without considering any interactions). Our goal was to explore a subset of all the possible models with different combinations of the variables in our hypothesis using the R package glmulti which uses a genetic algorithm for model exploration . We selected 10 combinations of the parameters conseq (5 and 10), sex rate (0.3, 0.4, 0.5, 0.6, and 0.7), and immigration rate (0.3, 0.4, 0.5, 0.6, and 0.7); the last two parameters are complementary and must add to one. Other parameters were left in their default values (popsize=100, deltaB=0, and deltaM=0.01), the mutation rate was set at 0.5, and the marginality parameter was set to TRUE, as this prevents the fitting of interaction terms. The confidence set of models to be generated after each genetic algorithm run (GAR) was set to 100 models.

Table S2. Set of variables and variable codings selected for estimating the relative importance in explaining the presence of *T. dimidiata* in households in Comapa and Zapotitlán, Jutiapa, Guatemala.

|  | **Variables** | **Variable Coding** |
| --- | --- | --- |
|  | **Ecological** |  |
| **1** | Altitude | <1100, 1100-1200, >1200 meters above sea level |
| **2** | Presence of coffee trees around the house | yes/no |
|  | **Biological** |  |
| **3** | Total number of humans in the house | 1-4, 5-8, >8 |
| **4** | Presence of cats | yes/no |
| **5** | Number of chickens owned | 0, 1-5, 6-10, >10 |
| **6** | Number of dogs | 0, 1-2, >2 |
| **7** | Armadillos seen by house owners | yes/no |
| **8** | Bats seen by house owners | yes/no |
| **9** | Opossum seen by house owners | yes/no |
| **10** | Animals present inside the house at night | yes/no |
| **11** | Animals present inside the house during the day | yes/no |
| **12** | *Rattus rattus* presence | yes/no (for a subset of houses only) |
| **13** | *Mus musculus* presence | yes/no (for a subset of houses only) |
| **14** | Chicken coop in peridomicile | yes/no |
|  | **Social** |  |
| **15** | Type of floor in the house | Dirt, other |
| **16** | Type of roof in the house | Metal sheet, other |
| **17** | Type of wall materials in the house | Adobe, bajareque, adobe-bajareque-other, block-brick, block-adobe-bajareque-other |
| **18** | Type of wall plaster in the house (interior ) | Complete, partial, no plaster |
| **19** | Type of wall plaster in the house (exterior) | Complete, partial, no plaster |
| **20** | Use of candles for house illumination | yes/no |
| **21** | Use of firewood in the house | yes/no |
| **22** | Number of rooms used to sleep | 1, 2, >2 |
| **23** | Number of persons per room | <3, 3-5, >5 |
| **24** | Cell phone ownership in the house | yes/no |
| **25** | Electricity in the house | yes/no |
| **26** | House age | <5, 6-10, 11-15, >15 years |
| **27** | Bug presence inside the house | yes/no (response variable) |

**Results**

Table S3. Socioeconomic factors from the Knowledge-Attitude-Practices and entomological survey showing association with triatomine infestation, as determined by the person-hour method (baseline survey, n=472).

| Variable | No. positive/Total no. (%) | Cramer´s *V* | Phi (*Φ*) | Odds Ratio  (95% CI) |
| --- | --- | --- | --- | --- |
| Ministry of Health household vector card present | 187/454 (41) |  | 0.115^b^ | 1.7 (1.1-2.7) |
| No. women <55 years old |  | 0.191^b^ |  |  |
| No. women >55 years old |  | 0.125^b^ |  |  |
| Bugs seen the last month |  | 0.193^b^ |  | 1.8 (0.8-4.2) |
| Vector control personnel have informed them about the disease | 52/241 (22) |  | 0.168^b^ | 2.5 (1.2-5.0) |
| Health Center has informed about the disease | 94/241 (39) |  | -0.164^b^ | 0.4 (0.2-0.8) |
| Prevents the disease with insecticide applications | 132/245 (54) |  | 0.106^a^ | 1.7 (0.9-3.3) |
| Access to piped water | 272/461 (59) |  | -0.089^a^ | 0.6 (0.4-1.0) |
| Access to electricity | 357/461 (77) |  | -0.116^b^ | 0.5 (0.3-0.9) |
| Uses the first surveyed room to sleep | 404/466 (87) |  | 0.111^b^ | 2.8 (1.2-6.7) |
| Uses the second surveyed room to sleep | 227/466 (49) |  | -0.107^b^ | 0.6 (0.4-0.9) |
| Uses the third surveyed room to sleep | 94/466 (20) |  | -0.130^b^ | 0.4 (0.2-0.8) |
| Uses the first surveyed room to store grain | 220/466 (47) |  | 0.097^a^ | 1.6 (1.0-2.5) |
| Uses candles for illumination | 111/469 (24) |  | 0.148^b^ | 2.2 (1.3-3.5) |
| Uses electrical lighting | 352/469 (75) |  | -0.141^b^ | 0.5 (0.3-0.8) |
| Uses propane gas for cooking | 99/469 (21) |  | -0.144^b^ | 0.3 (0.2-0.7) |
| Animals are kept in the house to prevent robbery | 61/459 (13) |  | 0.141^b^ | 2.4 (1.3-4.3) |
| Education level |  | 0.180^b^ |  |  |

^a^ Weak association, ^b^ Moderate association.

Table S4. Model results showing the least important (RI<0.75) individual factors to explain the relative odds of *Triatoma dimidiata* presence, keeping all other conditions constant.

| Factors  (odds_1_ / odds_2_) | Average Relative Importance (RI) | Average Weighted Estimate | Average Weighted Variance | Odds Ratio (95% Confidence Interval) |
| --- | --- | --- | --- | --- |
| Altitude 1100-1200/altitude <1100 | 0.49 | 0.00 | 0.03 | 1.00 (0.73-1.39) |
| Altitude >1200/altitude <1100 | 0.73 | 0.42 | 0.14 | 1.52 (0.73-3.20) |
| Animals inside night/no animals | 0.56 | 0.22 | 0.08 | 1.25 (0.72-2.16) |
| Animals inside day/no animals | 0.37 | -0.15 | 0.06 | 0.86 (0.53-1.38) |
| Armadillo yes/no | 0.19 | -0.04 | 0.02 | 0.96 (0.75-1.23) |
| Bats yes/no | 0.56 | 0.21 | 0.08 | 1.24 (0.72-2.13) |
| Candles yes/no | 0.39 | 0.11 | 0.04 | 1.11 (0.75-1.64) |
| Cats yes/no | 0.39 | 0.10 | 0.06 | 1.19 (0.67-1.81) |
| Cell phone yes/no | 0.15 | -0.03 | 0.01 | 0.97 (0.84-1.12) |
| Chicken coop yes/no | 0.24 | 0.01 | 0.01 | 1.01 (0.88-1.17) |
| 1-5 chickens/0 chickens | 0.03 | 0.01 | 0.00 | 1.01 (0.97-1.04) |
| 6-10 chickens/0 chickens | 0.03 | 0.00 | 0.00 | 1.00 (0.97-1.04) |
| >10 chickens/0 chickens | 0.00 | 0.00 | 0.00 | 1.00 (1.00-1.00) |
| Firewood yes/no | 0.30 | 0.01 | 0.01 | 1.01 (0.81-1.25) |
| Electricity in house yes/no | 0.14 | -0.03 | 0.01 | 0.97 (0.82-1.14) |
| House age 6-10 years/5 years | 0.01 | 0.00 | 0.00 | 1.00 (0.98-1.01) |
| House age 11-15 years/5 years | 0.01 | 0.00 | 0.00 | 1.00 (0.98-1.02) |
| House age >15/5 years | 0.08 | 0.01 | 0.00 | 1.01 (0.93-1.10) |
| Opossum yes/no | 0.35 | 0.05 | 0.02 | 1.05 (0.81-1.37) |
| 3-5 persons per room/<3 persons per room | 0.02 | 0.00 | 0.00 | 1.00 (0.99-1.01) |
| >5 persons per room/<3 persons per room | 0.02 | 0.00 | 0.00 | 1.00 (0.98-1.02) |
| 2 rooms used to sleep/ 1 room | 0.04 | 0.00 | 0.00 | 1.00 (0.97-1.03) |
| >2 rooms used to sleep/ 1 room | 0.04 | -0.01 | 0.00 | 0.99 (0.93-1.05) |
| Adobe walls/adobe-bajareque-other walls | 0.03 | -0.01 | 0.00 | 0.99 (0.94-1.05) |
| Bajareque walls/adobe-bajareque-other walls | 0.03 | -0.01 | 0.00 | 0.99 (0.92-1.06) |
| Block-brick walls/adobe-bajareque-other walls | 0.03 | -0.06 | 0.01 | 0.95 (0.75-1.19) |
| Block-adobe-bajareque-other walls/adobe-bajareque-other walls | 0.03 | -0.02 | 0.00 | 0.98 (0.88-1.08) |

Ratios indicate the two variables that are introduced in the model, after recoding the responses for each variable.

**S.References**

1. Votypka J, Synek P, Svobodova M (2009) Endophagy of biting midges attacking cavity-nesting birds. Med Vet Entomol 23: 277-280.

2. Schewe C, Weichert W, Dietel M (2011) Guidelines for molecular analysis in archive tissues; Stanta G, editor. Berlin, Heidelberg: Springer. 118 p.

3. Kent RJ, Thuma PE, Mharakurwa S, Norris DE (2007) Seasonality, blood feeding behavior, and transmission of *Plasmodium falciparum* by *Anopheles arabiensis* after an extended drought in southern Zambia. Am J Trop Med Hyg 76: 267-274.

4. Kent RJ, Norris DE (2005) Identification of mammalian blood meals in mosquitoes by a multiplexed polymerase chain reaction targeting cytochrome B. Am J Trop Med Hyg 73: 336-342.

5. Avila H, Goncalves A, Nehme N, Morel C, Simpson L (1990) Schizodeme analysis of *Trypanosoma cruzi* stocks from South and Central America by analysis of PCR-amplified minicircle variable region sequences. Mol Biochem Parasitol 42: 175-187.

6. Messenger LA, Llewellyn MS, Bhattacharyya T, Franzen O, Lewis MD, et al. (2012) Multiple mitochondrial introgression events and heteroplasmy in *Trypanosoma cruzi* revealed by maxicircle MLST and next generation sequencing. PLoS Negl Trop Dis 6: e1584.

7. Gaunt M, Yeo M, Frame I, Stothard J, Carrasco H, et al. (2003) Mechanism of genetic exchange in American trypanosomes. Nature 421: 936-939.

8. Calcagno V, Mazancourt C (2010) glmulti: an R package for easy automated model selection with (generalized) linear models. J Stat Softw 34: 1-29.
